# To Rezūm or Not to Rezūm: A Narrative Review of Water Vapor Thermal Therapy for Benign Prostatic Hyperplasia

**DOI:** 10.3390/jcm14124254

**Published:** 2025-06-15

**Authors:** Aris Kaltsas, Ilias Giannakodimos, Evangelos N. Symeonidis, Dimitrios Deligiannis, Marios Stavropoulos, Asterios Symeonidis, Konstantinos Adamos, Zisis Kratiras, Andreas Andreou, Michael Chrisofos

**Affiliations:** 1 Third Department of Urology, Attikon University Hospital, School of Medicine, National and Kapodistrian University of Athens, 12462 Athens, Greece; ares-kaltsas@hotmail.com (A.K.); iliasgiannakodimos@gmail.com (I.G.); d.delijohn@yahoo.gr (D.D.); stamarios@yahoo.gr (M.S.); constantinos.adamos@gmail.com (K.A.); zkratiras@gmail.com (Z.K.); 2 Department of Urology II, European Interbalkan Medical Center, 55535 Thessaloniki, Greece; evansimeonidis@gmail.com (E.N.S.); dr.andreou@yahoo.com (A.A.); 3 Department of Urology, Faculty of Medicine, School of Health Sciences, Aristotle University of Thessaloniki, 54124 Thessaloniki, Greece; symeaste@gmail.com

**Keywords:** benign prostatic hyperplasia, Rezūm therapy, water vapor thermal therapy, lower urinary tract symptoms, minimally invasive surgical therapies, Qmax, IPSS, prostate volume, sexual function, retreatment rate

## Abstract

**Background/Objectives**: Benign prostatic hyperplasia (BPH) is a common urological condition that can significantly impair quality of life in aging men by causing lower urinary tract symptoms (LUTS), including nocturia, weak stream, and incomplete emptying. While pharmacotherapy and surgical approaches such as transurethral resection of the prostate (TURP) remain cornerstone treatments, minimally invasive surgical therapies (MISTs) have emerged to bridge the gap between long-term medication use and invasive surgery. This narrative review assesses Rezūm therapy (water vapor thermal therapy, WVTT) by examining its mechanism of action, clinical efficacy, safety profile, and place in the BPH treatment algorithm. **Methods**: This narrative review synthesizes evidence from randomized controlled trials (RCTs), prospective studies, real-world cohorts, and published systematic reviews with meta-analyses to provide a comprehensive evaluation of Rezūm therapy for BPH. Key outcomes assessed include changes in International Prostate Symptom Score (IPSS), urinary flow rates, retreatment rates, adverse events, and sexual function preservation. **Results**: Across multiple studies, Rezūm significantly reduces IPSS (typically by ≥50%) and increases peak urinary flow by 4–5 mL/s. These improvements are durable, with five-year follow-up data showing low retreatment rates of approximately 4–5% and sustained symptom relief. The procedure, performed under local or minimal anesthesia, has a favorable safety profile: most adverse events are mild or transient, and notable complications, such as bleeding requiring transfusion or persistent sexual dysfunction, are rare. Importantly, Rezūm preserves both erectile and ejaculatory function in most patients, setting it apart from many traditional surgical interventions associated with higher sexual side effect rates. **Conclusions**: Rezūm is an effective and minimally invasive alternative for men with moderate prostatic enlargement who desire durable symptom improvement while avoiding the morbidity and sexual side effects associated with more invasive surgery. Future research should aim to further refine patient selection and assess long-term outcomes in broader populations.

## 1. Introduction

Benign prostatic hyperplasia (BPH) is a highly prevalent condition characterized by nonmalignant enlargement of the prostate gland that increases with age. Histological BPH is present in approximately 50–60% of men in their 60s, rising to 80–90% of men over 70 years [1]. Globally, over 210 million men were estimated to be affected by BPH as of 2010 [2]. Enlargement of the prostate, especially in the transition zone, can constrict the prostatic urethra and contribute to bladder outlet obstruction (BOO). In addition to mechanical obstruction, recent insights suggest that oxidative stress and chronic inflammation significantly contribute to BPH pathogenesis and may influence therapeutic outcomes [3]. This results in lower urinary tract symptoms (LUTS), such as urinary frequency, nocturia, weak stream, hesitancy, and incomplete emptying [4,5]. These symptoms can significantly impair quality of life and, if left untreated, may progress to acute urinary retention or recurrent urinary tract infections due to incomplete emptying [5]. Patho-physiologically, BOO in BPH has both a static component (physical compression of the urethra by the enlarged gland) and a dynamic component (increased smooth muscle tone within the prostate and bladder neck) [6]. Management of BPH aims to relieve obstruction, thereby improving symptoms and preventing complications [7].

Treatment options for BPH span a spectrum from conservative to surgical [8]. For mild symptoms, watchful waiting and lifestyle modification may be sufficient. Pharmacotherapy is the mainstay for moderate LUTS. Alpha-1 adrenergic blockers relax prostatic smooth muscle to address the dynamic component, typically improving symptom scores by a modest 4–6 points [9]. Additionally, 5-alpha-reductase inhibitors (5-ARIs) can shrink the prostate by 20% or more over several months by inducing prostatic epithelial atrophy, thereby reducing the static component of obstruction [10,11]. These medications can be effective, but long-term drug therapy may be associated with side effects (for example dizziness, sexual dysfunction) and adherence issues, and many patients have persistent symptoms despite combination medical therapy.

Surgical intervention is indicated when symptoms are severe, complications arise, or medical therapy fails. Transurethral resection of the prostate (TURP) has long been the gold-standard surgery for BPH, especially for prostate sizes up to about 80 mL [12]. TURP involves endoscopic resection of the hyperplastic tissue to create a wide channel, and it reliably alleviates obstruction with substantial improvements in urinary flow and symptom indices. However, TURP and other resective or enucleative surgeries (such as holmium laser enucleation of the prostate, HoLEP) carry risks inherent to more invasive procedures: bleeding, need for anesthesia or hospitalization, and a high rate of retrograde ejaculation due to disruption of the bladder neck (occurring in approximately 50–70% of TURP patients) [13]. In recent decades, various minimally invasive surgical therapies (MISTs) have been developed to bridge the gap between chronic medication and major surgery [14]. These include office-based or outpatient interventions designed to reduce prostatic obstruction with less morbidity. Notable MISTs for BPH include prostatic urethral lift (for example, UroLift^®^ implants), convective thermal therapies, Rezūm therapy (water vapor thermal therapy, WVTT), conductive thermal approaches, like microwave or radiofrequency ablation (TUMT, TUNA), and others, such as aquablation (water jet resection), intraprostatic stents or temporary devices (for example, iTIND), and prostatic artery embolization (PAE). Each of these seeks to improve LUTS while minimizing invasiveness and preserving quality of life, particularly sexual function [8,15].

Rezūm water vapor therapy is one such minimally invasive option that has gained traction in the last decade. It was approved by the United States FDA in 2015 as a novel therapy for BPH [16]. Given the increasing interest in therapies that offer a compromise between daily pills and surgery, it is essential to evaluate the role of Rezūm. This narrative review focuses on Rezūm therapy—its mechanism of action, clinical efficacy, safety profile, ideal patient selection—and discusses how it compares with other treatment modalities for BPH. We also highlight current evidence gaps and future directions for Rezūm in BPH management.

## 2. Search Strategy and Study Selection

This narrative review, rather than being a full systematic review or meta-analysis, aimed to capture a broad spectrum of literature pertaining to Rezūm therapy for BPH. To enhance transparency and replicability in accordance with peer review recommendations, a flexible search strategy was employed. PubMed/MEDLINE, EMBASE, Scopus, and the Cochrane Library were queried for English-language articles published from the inception of each database until February 2025.

Searches relied on combinations of Medical Subject Headings and keywords including “Benign Prostatic Hyperplasia,” “Water Vapor Therapy,” “Rezūm,” “Prostatic Hyperplasia,” “BPH,” “Minimally Invasive Surgical Therapies,” “Convective Thermal Therapy,” “Lower Urinary Tract Symptoms,” “LUTS,” “Prostate Volume,” “IPSS,” “Qmax,” “PVR,” and “IIEF-5.”

**Figure 1:**
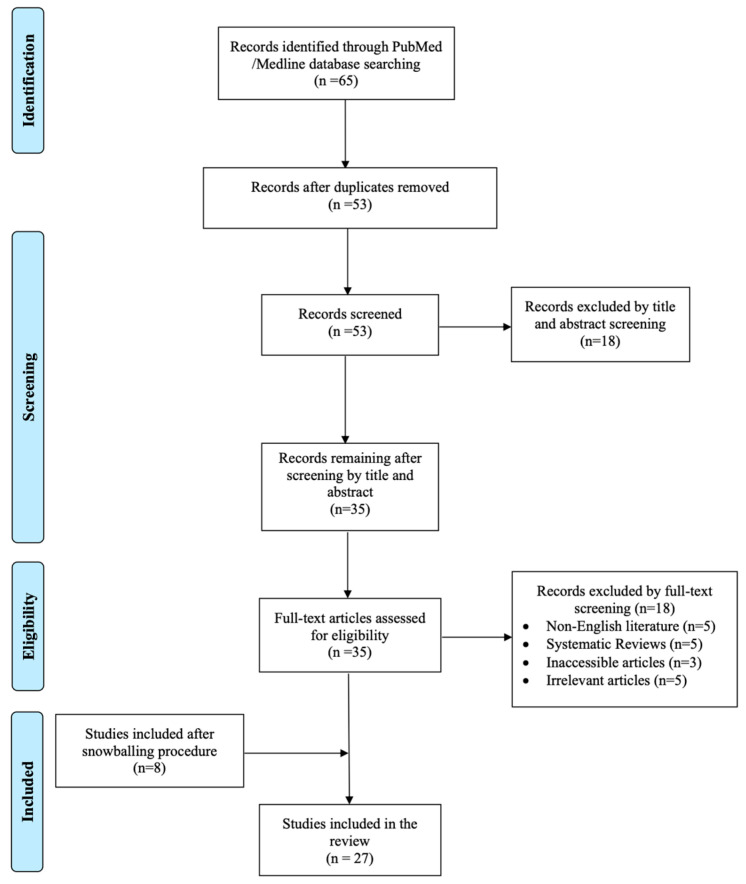
PRISMA Flow Diagram for Rezūm Therapy in Benign Prostatic Hyperplasia.

Titles and abstracts were initially screened for relevance, and the full text of potentially eligible articles was subsequently evaluated. Priority was given to randomized controlled trials comparing Rezūm to sham or other interventions, large prospective or retrospective cohorts enrolling more than 40 patients, systematic reviews that focused on Rezūm outcomes, and expert consensus documents or guidelines on water vapor thermal therapy. Basic science investigations without clinical endpoints, case reports, conference proceedings lacking full-text data, and non-English publications were excluded. Emphasis remained on synthesizing clinically pertinent information in a descriptive manner. Although this review is not a formal systematic review, a PRISMA-style flow diagram (Figure 1) has been adapted to illustrate the article selection process.

## 3. Mechanism of Rezūm Therapy

Rezūm therapy is a convective water vapor thermal therapy that ablates prostatic tissue by delivering thermal energy through injected steam. The Rezūm system is a handheld transurethral device that generate steam using radiofrequency energy delivered through a retractable needle [17,18]. During the outpatient procedure, the device is introduced via the urethra under endoscopic visualization. The small needle is deployed into prostatic lobes at prescribed locations, and a burst of steam is injected into the tissue, typically for ~9 s per lesion [17,18]. Each injection delivers a fixed volume of vapor (approximately 0.5 mL) that rapidly disperses through the tissue interstices. As the vapor condenses back to water within the prostate, it releases thermal energy (~70 °C) that causes immediate cell death in the targeted region via convective heating [19]. The thermal footprint of each injection is about 1–2 cm radius, creating a controlled necrotic lesion. Over the ensuing days to weeks, the ablated glandular tissue is resorbed or expelled, debulking the prostate and relieving urethral compression [20].

A key distinction of Rezūm compared to earlier thermal therapies for BPH lies in the mode of heat transfer. Convective heating (steam injection) can achieve rapid and uniform tissue destruction within a defined zone, whereas non-convective modalities, like transurethral microwave therapy (TUMT) or needle ablation (TUNA), rely on slower conductive heating that may be less consistent. In practice, Rezūm achieves tissue ablation without the need for active electrode heating or microwave antennae; this has simplified the procedure and improved patient tolerability. Notably, a recent network meta-analysis found that, at 12 months post-treatment, symptom and flow outcomes of Rezūm were comparable to those of legacy thermal therapies (TUNA, TUMT) [21], suggesting that convective water vapor achieves similar efficacy to these earlier modalities, albeit with modern advantages in delivery technique.

The Rezūm procedure is typically performed under local anesthesia (prostatic block) with oral or IV sedation, or alternatively under light general anesthesia, in an outpatient or office setting. The procedure duration is short; most treatments are completed in under 10 min, with multiple steam injections given across the prostate lobes. The number of injection sites is determined by prostate size and anatomy. For average moderate-size prostates (≈30–80 mL), around 4–10 injections are delivered, spaced from bladder neck to apex, including the median lobe if present [22]. In prostates larger than 80 mL, extended treatment covering lateral lobes in a distal to proximal fashion can be performed (often 10–15 injections), although this is off-label and requires operator experience [23]. Each vapor injection is placed approximately 1 cm apart to create overlapping zones of ablation. The convective heat effect is largely confined within the prostatic capsule, minimizing collateral damage to surrounding structures.

Compared to other ablative techniques, Rezūm’s mechanism offers some unique procedural advantages. It does not require energy delivery wires or fibers extending through the urethral wall (unlike TUNA’s needles or laser fibers), and the vapor penetrates effectively into tissue planes, including the median lobe. In contrast, prostatic urethral lift (UroLift) does not remove tissue at all but mechanically compresses it; laser vaporization or resection physically removes tissue but with more instrumentation and often in an operating room setting. Rezūm’s ability to treat the median lobe is a notable benefit—for instance, UroLift was initially not indicated for median lobes, whereas Rezūm is capable of ablating obstructive median lobe tissue by directing the steam needle into it [16]. The result is that Rezūm can address a wider range of anatomies, including tri-lobar hyperplasia, that might otherwise require resection.

After the Rezūm procedure, a temporary urinary catheter is usually placed, because the treated prostate becomes edematous and obstructive during the acute post-treatment phase. The convective thermal ablation causes an inflammatory reaction that peaks over the first week or two. Typically, the catheter is left in place for only a few days (often 3–5 days, depending on physician protocol and patient voiding trial) until the patient can urinate safely [24]. Over the subsequent weeks, necrotic tissue is gradually absorbed or expelled. Notably, symptom improvement with Rezūm is not immediate but rather gradual. The maximal therapeutic effect is usually observed at approximately 6 weeks to 3 months post-procedure, once significant tissue reduction has occurred [16]. This delayed full response contrasts with TURP or laser surgery, where debulking is immediate, but it is an expected aspect of convective thermal therapy. Patients should be counseled that urinary symptoms may actually transiently worsen in the days following Rezūm (due to swelling and irritation) before improvement manifests around the 1-month mark as healing progresses.

## 4. Clinical Efficacy

### 4.1. Symptom Relief and Voiding Improvements

Rezūm therapy has demonstrated significant efficacy in reducing LUTS severity in men with BPH. The pivotal evidence comes from a multicenter randomized controlled trial (Rezūm II trial), which compared Rezūm treatment to a sham procedure in men with moderate-to-severe symptoms (IPSS ≥ 13) and prostate volumes 30–80 mL. At 3 months, the Rezūm group showed marked improvement in symptoms compared to sham, and these benefits were sustained over time [25]. Final 5-year outcomes from this study showed that the IPSS was reduced by ~48% from baseline (a drop on the order of 11–12 points), and quality of life scores improved by 45% [25]. Peak urinary flow rate Qmax increased by approximately 4.4 mL/s on average, a 44% improvement from baseline [25]. These long-term data confirm that a one-time Rezūm treatment can produce durable relief of bladder outlet obstruction symptoms. Importantly, no significant loss of efficacy was observed between 1 year and 5 years in these trial patients—the symptom relief remained stable, indicating durable tissue effect [25].

Multiple other studies, including real-world cohorts, have reported similar magnitudes of improvement. In a large prospective series of 461 patients treated with Rezūm in the UK, the mean IPSS fell from about 21 at baseline to 5 at 12-month follow-up (a decrease of ~75%) [26]. Peak flow improved from 9.7 mL/s to 18.0 mL/s by 1 year [26], and post-void residual urine (PVR) decreased significantly, indicating better bladder emptying. Another single-center study of 193 patients noted a 46% reduction in IPSS and a 5 mL/s increase in Qmax at mean follow-up, along with a 50% reduction in residual urine volume [16]. Objective measures, such as urodynamic pressure-flow studies, also support Rezūm’s efficacy: in the subset of patients who underwent pre- and post-Rezūm urodynamics, the bladder outlet obstruction index (BOOI) fell by ~70%, confirming a clear reduction in outlet resistance [16]. These consistent outcomes across trials and clinical practice settings underscore that Rezūm meaningfully alleviates obstruction and improves voiding parameters for the majority of patients [25].

To provide an overview of the most recent and higher-level evidence supporting Rezūm’s effectiveness, a summary of meta-analyses is presented below (Table 1).

### 4.2. Durability of Effect

A critical consideration for any BPH therapy is how long the benefits last before re-intervention is needed. The available evidence indicates that Rezūm’s effects are durable at least in the mid-term (5 years). In the pivotal trial, the surgical retreatment rate was only 4.4% over 5 years [25]. In other words, 95% of patients remained free from needing additional invasive treatment (such as TURP or repeat Rezūm) through five years of follow-up. This low retreatment rate favorably compares to historical data for some other minimally invasive therapies. For instance, prostatic urethral lift implants have shown that approximately 13.6% of patients require re-intervention by 5 years [33], and prostatic artery embolization has reported over 20% retreatment within 2 years in trials [34]. Rezūm’s durability appears closer to that of TURP, which traditionally has about a 5–10% recurrence/retreatment rate at 5 years. The sustained symptom control and low retreatment frequency with Rezūm are attributed to its ability to ablate a significant volume of tissue (including median lobe tissue) and cause true anatomical debulking rather than just shifting prostatic tissue aside [24]. In a comparative context, a systematic review concluded that Rezūm’s need for re-intervention up to 3 years was statistically similar to TUMT and TUNA, and not substantially inferior to more aggressive surgeries [21]. Real-world registry studies have also corroborated a low re-treatment incidence: one multicenter report found that retreatments tend to occur early (within the first 1–2 years) if at all, and plateau thereafter [26].

Recent real-world registry data in 712 patients (mean prostate ~74 mL) confirm sustained symptom relief out to 3 years, with IPSS decreasing from 22 to ~10 at 1 year and remaining stable through year 3 [35,36]. Objective measures, such as Qmax, also show persistent improvement (increasing from ~8.6 to 14.5 mL/s at 12 months, with benefits maintained at 3 years) [35]. Experts underscore that the short-term inconvenience of post-procedure dysuria and catheterization is justified by these durable outcomes, as many men perceive the transient discomfort as a favorable trade-off for prolonged relief [35,36].

### 4.3. Symptom Onset and Timeline

**Figure 2:**
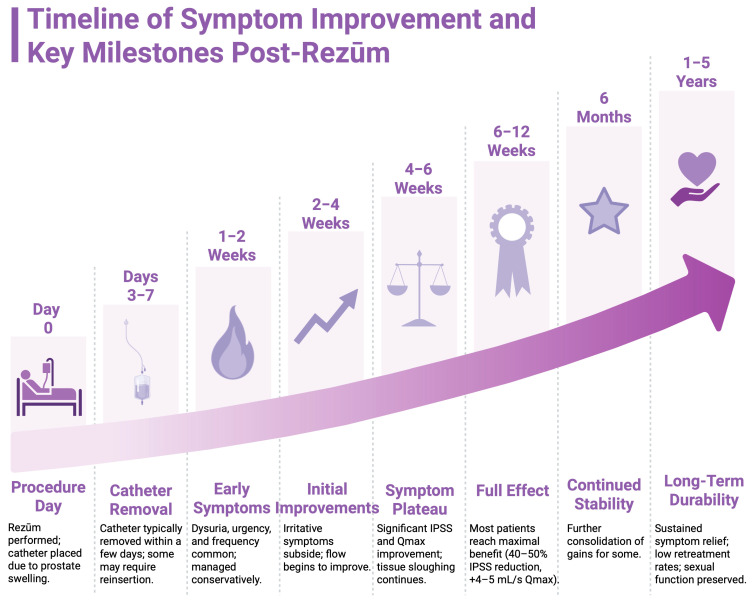
Timeline of symptom improvement and key milestones following Rezūm therapy. Created in BioRender. Kaltsas, A. (2025) https://BioRender.com/ujpdmx4.

Patients treated with Rezūm usually experience noticeable symptom improvement by 1–3 months post-procedure. The randomized trial noted significant symptom score differences as early as the first follow-up (<3 months) [25]. In a real-world context, by 3 months post-Rezūm, average IPSS reductions of around 10 points and flow rate increases are seen [26]. The improvement often continues or is maintained at 6 and 12 months as the full effect materializes. Unlike surgery, which has immediate anatomical effect, Rezūm’s timeline requires patience during the initial healing. Clinicians generally consider a successful outcome by 3 months, with incremental gains possible up to 6 months as residual tissue sloughs off. Durability beyond the first year is excellent, with 2-year, 3-year, and 4-year data from the original trial showing persistence of the initial benefits [37]. Another analysis specifically focused on sexual function outcomes confirmed that the preservation of function and symptom relief remained stable through the 5-year mark, reinforcing the long-term therapeutic stability of Rezūm [25]. A visual summary of the expected postoperative course and symptom trajectory following Rezūm therapy is presented in Figure 2.

### 4.4. Influence of Prostate Size

Rezūm was initially studied in prostates 30–80 mL, and guidelines have generally confined its routine use to this range [38]. There is growing interest in using Rezūm for larger prostates (≥80–100 mL) to offer a less invasive alternative to enucleation or open surgery in select patients. Emerging data suggest that Rezūm can be applied to larger glands with some modifications and with reasonable short-term outcomes. In a retrospective comparison, men with prostates ≥ 80 mL (mean ~107 mL) treated with Rezūm had similar symptomatic improvement as those with smaller prostates after 6–12 months [36]. The IPSS and flow rate gains were comparable, indicating that even large glands responded to steam ablation. However, there were some notable differences: patients with very large prostates experienced longer postoperative catheterization (on average 9 days vs. 6 days for smaller prostates) and a higher incidence of post-procedure urinary tract infection or urosepsis (5.6% vs. 0% in one series) [36]. Additionally, the retreatment rate at 1 year was higher in the large prostate group (8.3%) compared to the standard-size group (4.8%), although this difference did not reach statistical significance in that study [36]. These findings suggest that, while Rezūm can debulk large prostates to a degree, the risk of complications and need for additional therapy may increase when prostate size exceeds ~80 mL. Ongoing studies are evaluating protocol adaptations (such as staging the procedure or combining with adjunctive measures) to improve outcomes in large glands. Until more data are available, most clinical guidelines consider Rezūm suitable primarily for small-to-moderate size prostates, and careful patient counseling is needed if using it for glands significantly over 80 mL [36]. If a patient with a large prostate is unfit or unwilling for surgery, Rezūm could be considered as a temporizing measure or partial debulking, with the understanding that additional therapy may be needed down the line [39].

**Figure 3:**
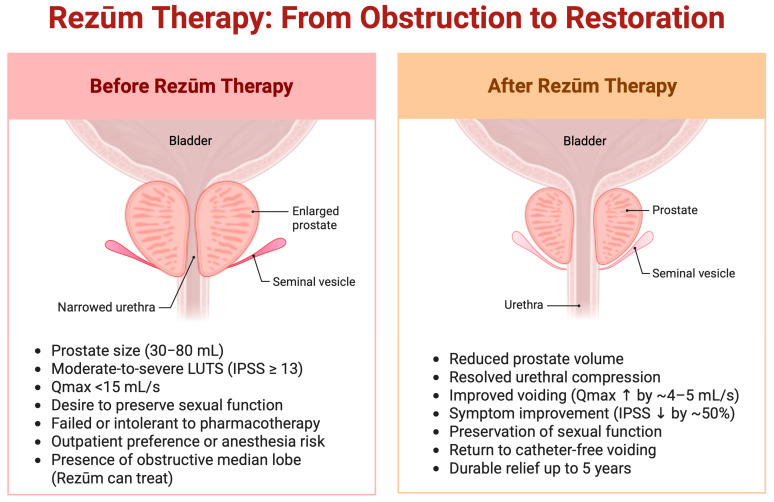
Coronal views of the prostate before and after Rezūm therapy. Created in BioRender. Kaltsas, A. (2025) https://BioRender.com/56b0nsk.

Newer evidence indicates that Rezūm may be effective for prostates ≥ 80 mL. A recent series in 36 men (mean volume ~107 mL) reported postoperative voiding improvements and IPSS reductions comparable to prostates < 80 mL [35,36]. Although alpha-blocker usage declined similarly between groups (from ~94% to 61% in large prostates), catheterization lasted longer (9 vs. 5.7 days), and urosepsis, while infrequent (5.6%), occurred only in the large-prostate cohort [16,36]. Expert panels now endorse Rezūm for moderate-to-severe LUTS even if volume exceeds 80 mL, contingent on careful technique and extended catheterization (10–14 days) [35,36]. Figure 3 provides a coronal visual comparison of the prostate before and after Rezūm therapy, illustrating key anatomical and clinical changes associated with treatment.

## 5. Safety and Adverse Effects

One of the attractive features of Rezūm therapy is its favorable safety profile compared to more invasive surgeries. The procedure avoids cutting or excising tissue in real-time, which greatly reduces intraoperative bleeding and eliminates the risks of dilutional hyponatremia (“TUR syndrome”) associated with TURP. In general, Rezūm is considered a low-risk outpatient procedure, and most adverse events are mild, transient, and related to the post-treatment inflammatory response [40].

### 5.1. Common Postoperative Symptoms

Nearly all patients experience transient urinary symptoms following Rezūm as the ablated tissue causes inflammation. These symptoms include dysuria (painful urination), urinary frequency and urgency, hematuria (blood-tinged urine), and pelvic discomfort. In the pivotal RCT and other studies, irritative urinary symptoms are commonly reported in the first few weeks. For example, dysuria and frequency can occur in a significant subset of patients (20–40%) during week 1–2 post-procedure, but these usually resolve over time with conservative management (hydration, NSAIDs, alpha-blockers for bladder spasm). Minor hematuria is expected as necrotic tissue sloughs off; patients may note reddish or debris-filled urine for several days [40]. Infectious complications like urinary tract infection (UTI) can occur but are relatively infrequent. In a series of high-risk older patients, the UTI rate post-Rezūm was around 3.9%, and minor hematuria occurred in 4.4% [41]. Prophylactic antibiotics around the time of the procedure are commonly given to minimize infection risk. Urinary retention requiring recatheterization can happen if post-procedure swelling is pronounced; even with routine catheter placement at the time of Rezūm, a small percentage of patients may fail their voiding trial and need a catheter reinserted for a few extra days. However, in most cases the catheter is successfully removed within a week and any retention is temporary. Real-world data indicate that by one month post-Rezūm, the vast majority of men are catheter-free and voiding well [41].

Serious adverse events are uncommon with Rezūm therapy. Since the procedure does not involve cutting or resection of prostatic tissue, bleeding requiring transfusion is essentially absent—unlike TURP, which carries an approximate 2% transfusion risk in patient with large glands. Urosepsis, a severe infection that enters the bloodstream, has been reported rarely following Rezūm therapy, primarily in patients with very large prostates or those requiring prolonged catheterization. In one comparison, about 5.6% of men with prostates ≥ 80 mL developed urosepsis post-Rezūm versus none in the smaller prostate group [36]. This suggests that treating extensive tissue volumes might slightly elevate infection risk, possibly due to more necrotic load or longer catheter dwell time. Nonetheless, overall rates of severe infection remain low. Urethral stricture or bladder neck contracture as a result of Rezūm are also rare, given that the procedure does not involve resection or cautery along the urethral circumference. Long-term urinary incontinence has not been reported as a direct complication—the external urinary sphincter is distal to the treatment area and is not affected by the steam injections [14].

In a multi-center real-world analysis of 461 patients (mean follow-up ~17 months), the 4.6% surgical retreatment rate (2.4% in the first year) paralleled the 5-year RCT figure of 4.4%, reinforcing reproducible outcomes [25,26]. These later studies noted that retreatments were commonly tied to unaddressed median lobe or suboptimal bladder-neck management rather than device malfunction [26]. Major complications remain rare, and a single-center cohort of 193 reported zero intraoperative adverse events or Clavien–Dindo ≥ 3 complications [16]. Large datasets further highlight lower reintervention with Rezūm (6.8%) than UroLift (10.8%) at 5 years, albeit with higher transient urinary retention (~23%) [42].

### 5.2. Sexual Function Outcomes

A major advantage of Rezūm over many surgical therapies is the preservation of sexual function, particularly ejaculatory function. Because Rezūm does not mechanically remove or damage the structures of the bladder neck and prostate apex that are involved in ejaculation, the risk of retrograde ejaculation is very low. In the 5-year trial, there were “no reports of device or procedure related sexual dysfunction or sustained de novo erectile dysfunction” [25]. Erectile function (as measured by IIEF scores) remained statistically unchanged from baseline through 5 years for Rezūm-treated men, and importantly, ejaculatory function was preserved in the vast majority. In a dedicated 5-year analysis of sexual health outcomes, Rezūm therapy did not worsen mean ejaculatory dysfunction scores; men continued to have antegrade ejaculations in contrast to the high rates of anejaculation seen with TURP [25]. These findings align with earlier 3-year data showing no significant change in erectile or ejaculatory function compared to sham controls [14]. By avoiding damage to the nerves and muscular fibers critical for sexual function, Rezūm offers an option for sexually active men who wish to minimize the risk of impotence or ejaculatory loss. Recent literature emphasizes the importance of sexual health in surgical decision-making for BPH, highlighting that minimally invasive techniques like Rezūm may strike a favorable balance between symptom control and preservation of erectile and ejaculatory function [43]. This is a significant consideration, as traditional surgeries like TURP and laser prostatectomy commonly result in retrograde ejaculation in 50–75% of patients [44]. Even newer surgeries like HoLEP have reported retrograde ejaculation rates of around 30–40% or higher, depending on technique. Therefore, Rezūm’s ability to relieve obstruction while preserving ejaculation is a key benefit for quality of life. It should be noted that, while most men maintain normal ejaculation after Rezūm, a small number might experience some reduction in ejaculate volume or a transient change in sexual function due to post-procedure discomfort. However, unlike resective surgeries, Rezūm is not intrinsically expected to cause permanent ejaculatory dysfunction.

A 4-year real-world follow-up of 91 patients found no de novo erectile dysfunction in men previously potent; some with baseline ED even noted a 30% rise in erectile function scores post-Rezūm [45]. This improvement may partly stem from discontinuing BPH medications that negatively impact sexual health. Younger men may particularly benefit from Rezūm’s negligible effect on ejaculation—studies quote ~3% retrograde ejaculation, which is near baseline incidence [45].

### 5.3. Recovery and Return to Activity

Recovery after Rezūm is generally quick in terms of resuming normal activities, but symptom recovery is gradual. Most patients can return to light daily activities within a day or two after the procedure, given that there are no surgical wounds or significant systemic effects [20]. They will typically have a catheter for several days, which can be a minor hindrance but not debilitating. Once the catheter is removed, patients might still experience urinary urgency or slight discomfort for a couple of weeks. By 2–4 weeks, many of the acute symptoms subside. By contrast, after TURP, although the obstruction is removed, patients often need a few weeks for the bleeding to settle and for the urinary tract to heal from resection, during which they may see blood in urine or experience frequency. Rezūm patients usually do not have the prolonged bleeding or need for irrigation that TURP patients have in the immediate postoperative period. Thus, “social” recovery (return to work, etc.) is often faster with Rezūm—many patients are able to resume work within a week if their job is not physically strenuous, versus 2–4 weeks after TURP. As noted, the full symptomatic improvement with Rezūm is slower; patients might still benefit from continued use of an alpha-blocker for a month or so after the procedure to ease symptoms during healing [37]. By about 3 months, the maximum benefit is realized in most cases [16], and at that point the recovery can be considered complete.

## 6. Patient Selection and Considerations

Appropriate patient selection is crucial for optimizing Rezūm outcomes. The ideal candidate for Rezūm is a man with moderate to severe LUTS from BPH who desires symptom relief beyond what medications can provide, but who also wishes to avoid or defer the morbidity of traditional surgery. Factors such as the patient’s symptom severity, bother level, prostate anatomy, comorbidities, and personal preferences (regarding factors like anesthesia, recovery time, and sexual side effects) should determine the optimal patients for Rezūm. However, within this broad context, several specific considerations should guide selection.

### 6.1. Medical Comorbidities and Surgical Risk

Rezūm can be an excellent option for patients who are poor candidates for surgery under anesthesia. The procedure can be carried out with minimal sedation, even in an office setting, which is advantageous for older patients with multiple comorbid conditions (cardiac, pulmonary, etc.). Rezūm offers a chance to relieve the obstruction in such “frail” patients without subjecting them to major surgery. A multicenter study in multimorbid, catheter-dependent patients (mean age ~80) demonstrated that Rezūm could successfully eliminate the need for chronic catheterization in about 79% of such high-risk individuals, allowing them to void naturally again [41]. This indicates that even patients deemed “unfit for surgery” can be considered for Rezūm, as it is significantly less invasive. That said, one must consider life expectancy and goals of care in very elderly patients; if their symptom burden is low or they have other priorities, an intervention might be deferred. Conversely, younger, healthier men can also be candidates if they want to avoid the permanent side effects of TURP. Minore et al. demonstrated encouraging early outcomes for patients < 50 years old undergoing WVTT, with all of them maintaining or gaining antegrade ejaculation [46]. Additionally, younger patients with BPH often have relatively smaller prostates and vigorous healing, which makes them particularly well-suited to an intervention like Rezūm.

### 6.2. Contraindications and Special Considerations

Certain patients should not undergo Rezūm or should do so only with special consideration. Those with obstructive median lobes can be treated but, if the median lobe is very large and calcified (a “stone” prostate), results may be less optimal. Men with prior prostate or urethral surgery (for example, prior TURP, urethral stricture repair) are usually excluded in trials, but in practice Rezūm has been carried out as a repeat procedure for regrowth after TURP or to treat recurrent obstruction—success in these cases can vary. Active urinary infection is a contraindication; any UTI should be treated before Rezūm to reduce the risk of sepsis. Patients with suspected or known prostate cancer generally should not receive Rezūm without thorough evaluation, as the presence of cancer could be missed while treating BPH. PSA-based diagnostics remain essential in this context, though their specificity for cancer versus benign disease remains limited and should always be interpreted in conjunction with clinical and histological findings [47]. Lifestyle factors, such as alcohol consumption, may modulate prostate cancer risk and should be considered in comprehensive assessments prior to BPH intervention [48]. In addition, patients with gross hematuria from other causes (bladder tumors, etc.) or significant bladder dysfunction (for example, atonic bladder) will not benefit from Rezūm, since their issues lie outside the prostate. Neurological conditions affecting voiding (neurogenic bladder) were excluded from most Rezūm studies [16], so outcomes in those groups are not well-defined.

## 7. Comparative Effectiveness of Rezūm

Minimally invasive Rezūm therapy occupies an intermediate position among BPH treatments. It is helpful to contrast Rezūm’s outcomes and trade-offs with other available therapies.

### 7.1. TURP (Transurethral Resection of the Prostate)

TURP is the benchmark for symptom relief, typically yielding the greatest and quickest reduction in obstruction. TURP can decrease IPSS by ~70–80% and often doubles or triples the peak flow rate. In direct comparisons, Rezūm’s improvements in symptoms and flow are substantial but somewhat less than what a successful TURP achieves. However, this comes at the cost of higher invasiveness. TURP requires regional or general anesthesia, an operating room setting, and usually an overnight hospital stay. The complication profile of TURP includes bleeding, a need for irrigation catheters, and a significant risk of sexual side effects (ejaculatory dysfunction in 50–70% of cases) [13]. In contrast, Rezūm is an outpatient procedure under minimal anesthesia, with virtually no bleeding and preservation of antegrade ejaculation in most patients [14]. Rezūm also has a lower risk of postoperative complications, like strictures. The flip side is that TURP provides immediate obstruction relief (once healing is over in a couple weeks), whereas Rezūm requires patience for results over 1–3 months [17,18]. Regarding durability, TURP has a long track record of lasting >10 years for many patients, though regrowth can occur; Rezūm has proven durability to 5 years and counting. Notably, the 5-year retreatment rates (≈5% for Rezūm [25]) are not drastically different from TURP’s retreatment rates in contemporary series (perhaps 1–2% per year cumulative). Thus, for moderate-size prostates, Rezūm offers a less invasive alternative to TURP with nearly comparable mid-term efficacy. TURP might still be preferred for very large prostates where a single session of Rezūm might not remove enough tissue, or in cases where patients require the absolute fastest relief. However, for many men, Rezūm can achieve the needed relief without the downsides of TURP. This has led some authors to suggest that WVTT can serve as a first-line treatment for appropriate patients, reserving TURP only for those who fail a minimally invasive approach [25].

### 7.2. Prostatic Urethral Lift (UroLift)

UroLift is another popular office-based therapy that involves implanting small permanent clips to retract prostatic lobes and widen the urethral lumen. In terms of outcomes, UroLift and Rezūm are both considered “minimally invasive,” but they differ in mechanism and profile. UroLift tends to have an immediate effect—patients often notice improvement within 2 weeks, since no tissue needs to heal; the prostate is simply held open by the implants. Rezūm’s effects, as noted, are delayed for a few weeks. UroLift’s symptom score improvements average around 35–40% reduction in IPSS and a modest improvement in flow (Qmax increases ~3–4 mL/s) at 1 year. Rezūm’s improvements in IPSS are often greater (~50% reduction or more) [25], and flow rate improvements are somewhat larger on average (because actual tissue is removed in the long run). A key distinction is durability and retreatment: 5-year data for the UroLift device show a retreatment rate of about 13.6% [33], which is higher than the ~4–5% seen with Rezūm [26]. This suggests that Rezūm may achieve a more permanent reduction in obstruction. UroLift’s unique selling point is that it causes virtually no sexual dysfunction as well—it avoids ejaculatory issues, just like Rezūm (since it does not affect the bladder neck). Both procedures are typically performed with local anesthesia in an outpatient setting. UroLift does not require a post-op catheter in most cases, whereas Rezūm usually does for a few days. UroLift’s side effects include temporary pelvic pain, urgency, or hematuria, but generally patients recover very quickly (within days). Rezūm’s side effects (dysuria, urgency) can last longer in the initial weeks. Another practical consideration is that UroLift leaves behind permanent implants in the prostate, which can be a concern if the patient later needs an MRI (the clips are MRI-safe but can cause artifact) or another procedure. Rezūm leaves no foreign material behind. Cost-wise, the two are similar per procedure, though UroLift cost accumulates with each implant used (usually 4–6 implants). A network meta-analysis indirectly comparing various MISTs found Rezūm and UroLift (PUL) both improved symptoms significantly over sham, with Rezūm perhaps offering slightly greater IPSS reduction by 1 year, but each has advantages and neither causes appreciable erectile dysfunction [24]. In direct comparisons with UroLift, Rezūm has demonstrated lower 5-year retreatment (6.8% vs. 10.8%), yet tends to cause earlier irritative symptoms [42]. In practice, both are good options for men with prostates in the 30–70 mL range, and the presence of a median lobe might tilt the decision in favor of Rezūm. An extended 4-year Medicare model showed Rezūm dominance over UroLift, yielding more quality-adjusted life years (QALYs) (3.548 vs. 3.490) at lower cost [49].

### 7.3. HoLEP (Holmium Laser Enucleation of the Prostate)

HoLEP is a transurethral endoscopic laser surgery that can remove the entire adenomatous portion of the prostate, even in very large glands (>100 mL). It has been replacing open prostatectomy in many centers. Compared to Rezūm, HoLEP is a much more invasive procedure—it requires general or spinal anesthesia, an operating room, and significant endoscopic surgical skill. Its outcomes in terms of symptom relief are excellent, often equivalent to a complete TURP or open removal regardless of prostate size. HoLEP can achieve very high flow rates and very low post-op symptom scores and is considered the definitive therapy for large prostates. However, HoLEP also commonly results in retrograde ejaculation (rates vary 30–80% depending on technique) because it removes tissue around the verumontanum and internal sphincter. The risk of incontinence is low but slightly higher than TURP in inexperienced hands (due to the possibility of sphincter injury, though in experienced hands long-term incontinence is <2%). The recovery from HoLEP is on par with TURP—a catheter for ~1 day and some irritative symptoms for a few weeks due to the large cavity created. When comparing Rezūm to HoLEP, they really serve different populations: HoLEP is ideally suited for men with very large prostates or those who want a “one and done” solution and don’t mind a more involved procedure, whereas Rezūm is for those who prioritize minimal invasiveness and are within a moderate prostate size range. Cost-wise, HoLEP in a hospital is more expensive upfront than an office Rezūm procedure.

In terms of long-term results, HoLEP’s are proven to be durable (10+ years data) and Rezūm is. so far, durable to 5 years in studies. HoLEP also provides tissue for pathological analysis (to rule out incidental prostate cancer), whereas Rezūm vaporizes tissue in situ (no tissue is removed for pathology). For a patient concerned about missing a cancer diagnosis, HoLEP or TURP has that advantage. Overall, HoLEP remains the gold standard for large-volume BPH and maximally effective debulking; Rezūm is not competing in that space, so much as it is providing an alternative for smaller glands or those who will not accept the side effects of resection [50].

Matched-pair data show HoLEP yields larger IPSS and Qmax improvements—especially in bigger prostates—but Rezūm outperforms in preserving ejaculation and offers fewer complications [51]. Indeed, patient satisfaction can remain high with Rezūm despite slightly lesser symptom relief, given fewer sexual side effects [51].

### 7.4. Pharmacotherapy (Medications)

Many patients facing a decision between Rezūm and other therapies will have already been on medications like alpha-blockers and 5-ARIs. Comparing Rezūm to continued medical therapy is important from a patient and health system perspective. Medications can certainly improve symptoms, but typically by a more modest degree (~4–6 point IPSS improvement for alpha-blockers [9], and an additional few points if 5-ARIs are added over months). Rezūm’s average improvement (~11–15 points IPSS) clearly exceeds what most patients achieve on drug therapy [52]. Thus, for symptom severity, Rezūm can offer a greater payoff. Additionally, medications must be taken daily indefinitely, with cumulative side effects: alpha-blockers can cause dizziness, orthostatic hypotension, fatigue, and ejaculatory dysfunction (though typically less severe than surgery-related EjD), and 5-ARIs can cause sexual side effects (erectile dysfunction, loss of libido, ejaculatory reduction) in a subset of men [53]. Rezūm, as a one-time treatment, avoids the need for daily pills and the systemic side effects that come with them. From a cost standpoint, while Rezūm has an upfront cost, it may become cost-effective over time compared to buying medications for years. A model-based cost-effectiveness analysis found that minimally invasive therapies like Rezūm provided more QALYs at a lower lifetime cost than continuing pharmacotherapy, for moderate-to-severe BPH patients [54]. In that study, initial treatment with WVTT yielded the highest QALYs and the lowest projected cost, dominating prolonged drug therapy in the long run [54]. Many men on medications will eventually progress or remain symptomatic and need some intervention; performing Rezūm earlier could spare them years of drug side effects and expense. The tipping point is often when symptoms are no longer acceptably controlled or the burden of taking multiple medications (and dealing with their side effects) outweighs the perceived risk of a procedure. In summary, compared with medical therapy, Rezūm provides significantly greater improvement in LUTS and freedom from daily drug burden, at the cost of a one-time minimally invasive procedure. Current trends and guidelines suggest that men who are good candidates for MISTs and are dissatisfied with medications should be offered therapies like Rezūm as a viable alternative to simply adding more medications or moving straight to TURP [37].

Sensitivity analyses upheld Rezūm’s cost advantage in ≥99% of simulations. Meanwhile, a Canadian study demonstrated higher lifetime QALYs and reduced cumulative expenses when initiating Rezūm earlier rather than continuing medications [55].

## 8. Future Directions

As Rezūm therapy becomes more widely adopted, several avenues for future research and development are emerging to further define its role in BPH management.

### 8.1. Long-Term Outcomes

While we now have solid 5-year data demonstrating durable symptom relief and low retreatment rates [25], longer-term follow-up will be important to see how Rezūm holds up at 7, 10, or even 15 years post-treatment. BPH is a chronic progressive condition, and it is possible that regrowth of prostate tissue or new hyperplasia could occur over a long time horizon, potentially necessitating re-intervention. Ongoing extension studies of the initial Rezūm trial cohort are likely to report results beyond 5 years in the coming years. Long-term data will also inform whether Rezūm simply delays eventual more invasive therapy for some, or if it can truly serve as a lasting definitive treatment in a large fraction of patients. The current 5-year results are very encouraging (with ~95% of men avoiding surgery in that period) [25] so, if similar efficacy is seen at 8–10 years, Rezūm’s status as a durable solution will be cemented.

### 8.2. Use in Larger Prostates

Future prospective trials could clarify the safety and optimal technique for treating prostates of larger volumes, such as 80–150 mL. It may be that modifications, like performing Rezūm in two stages (treating one lobe at a time to reduce acute swelling) or combining Rezūm with an immediate bladder neck incision (to assist voiding preemptively) could improve outcomes for big prostates [26]. Indeed, some practitioners have reported using a concurrent bladder neck incision along with Rezūm in men with tight bladder necks or smaller prostates, to mitigate obstruction during the healing phase [26]. Investigating such combination approaches formally would be valuable. Additionally, engineering advances in the Rezūm device might occur—for example, a higher volume or longer steam injection for large glands, or a modified protocol for very tall prostates [23]. As evidence builds, guidelines may broaden the recommended prostate size range. The NICE guidance currently indicates limited evidence for volumes of ≥80 mL [26]; however, with additional data, Rezūm may become an accepted option for volumes up to 100 mL or more. This would not only apply to a specific subset of patients, such as those who are not candidates for major surgery or who are unwilling to undergo it, but also to a broader group of patients.

### 8.3. Combination with Medical Therapy

Another future consideration is how Rezūm fits into combination therapy strategies. One might envision using 5-ARIs after Rezūm to potentially enhance prostate shrinkage, or continuing alpha-blockers for a while to ease the irritative symptoms. In current practice, many urologists continue alpha-blocker therapy for several weeks post-Rezūm to help patients through the healing phase, stopping it once the Rezūm effect has kicked in. Research into whether pre-treating with a 5-ARI (to shrink the prostate a little and perhaps reduce post-treatment swelling) could improve Rezūm outcomes would be interesting, especially for larger prostates [10,53]. There is also the scenario of combination with other procedures: for instance, using Rezūm to treat lateral lobes and UroLift implants to retract a particularly large median lobe or vice versa, though such hybrid approaches are largely hypothetical at this point.

### 8.4. Guideline Integration

As evidence accumulates, we expect major urological guidelines to further incorporate Rezūm. The American Urological Association (AUA) guidelines (updated in 2021 and amended 2023) already list WVTT as an option for men with appropriate prostate sizes and symptom severity, alongside other MISTs and standard surgeries [56]. The European Association of Urology (EAU) has been more cautious, noting that Rezūm is still relatively new and listing it as an “investigational” option pending longer-term results [57]. With 5-year data now published and more international experience, it is likely that European guidelines will formally endorse Rezūm as a recommended treatment for LUTS/BPH in upcoming updates, perhaps with caveats on prostate size. Beyond these, an international consensus in 2025 highlights that Rezūm can be considered first-line in well-selected men, including those with median lobes, provided technique accounts for thorough ablation [22]. Future guidelines may also refine which patient profiles are best served by Rezūm.

### 8.5. Technological and Procedural Advances

On the technology side, Rezūm’s current system could be improved in small ways—perhaps with a more ergonomic device, or integration with imaging (to target injections more precisely). One challenge sometimes noted is the proper placing of injections in very large median lobes or in unusual anatomies; better visualization or even robotic assistance could be areas of future innovation. Another area is patient comfort during the procedure: techniques like using a periprostatic nerve block (as done in transrectal biopsy) to numb the prostate have been employed to make office Rezūm more tolerable, allowing some procedures to be performed under local anesthesia without heavy sedation [41]. Studying the optimal anesthesia method (local vs. sedation vs. general) for patient experience would be useful.

Another well-established point to consider is that a surgeon can achieve significant linear improvements in surgical and functional outcomes after a series of consecutive cases, even during the early phase of a learning curve (LC) [58]. Although relatively easy to perform and without a steep LC, to date, there is currently no formal analysis indicating a level at which a surgeon achieves adequate results to draw consistent conclusions for Rezūm. Clarifying this concept in conjunction with its influence on perioperative parameters is essential. Besides, it appears that Rezūm can be successfully performed during the early LC with an acceptable safety and efficacy profile. Rezūm simulators can be a critical step in this direction and potentially revolutionize the method’s adoption in the future [59]. A prospective sedation comparison indicated that oral sedation plus local anesthesia (“OSLA”) is feasible, minimizing anesthetic risk and lowering costs [60].

## 9. Conclusions

BPH remains a significant clinical challenge due to its widespread occurrence and its profound effects on the quality of life in older men. Among the available treatment modalities, Rezūm water vapor therapy has emerged as a valuable minimally invasive approach. Rezūm provides substantial symptom relief while avoiding many of the risks associated with more invasive surgical options, such as TURP. Its favorable safety profile, including minimal adverse effects and the preservation of sexual function, makes it particularly attractive to patients seeking a balance between efficacy and quality of life. Given these characteristics, Rezūm represents an important therapeutic option, positioned between long-term pharmacological management and traditional surgical interventions.

Future research will help clarify the optimal use of Rezūm therapy, particularly regarding its role in managing larger prostate sizes and refining patient selection guidelines. Comparative studies against traditional surgical and emerging minimally invasive techniques will also help delineate its precise position within the treatment algorithm for BPH. Ultimately, “To Rezūm or not to Rezūm” is best decided through shared decision-making, where patient preferences, comorbidities, and anatomical factors guide individualized treatment planning. In an era emphasizing both efficacy and quality of life, Rezūm’s proven track record for sustained symptom relief, low complication rates, and sexual function preservation secures its place in the evolving landscape of BPH management.

## Figures and Tables

**Table 1:** Up-to-date overview of meta-analyses on WVTT using the Rezūm system for treating LUTS related to BPH.

Authors, Year (Reference)	Studies (Patients)	IPSS Change	Qmax Change	PVR Change	Void Volume (VV)	IIEF-5 Change	Main Conclusions
Yang et al., 2025 [27]	16 RCTs (1622)	Similar to TUNA/TUMT	Similar to TUNA/TUMT	Comparable/ no advantage	NR	No adverse effect, minimal EjD	Rezūm comparable to TUNA/TUMT; minimal sexual side effects.
McVary et al., 2024 [28]	15 (471)	−11.0 pts	+6.5 mL/s	−101 mL	NR	No significant change	Effective in large prostates; minimal complications and retreatment.
Çakiroğlu et al., 2023 [29]	21 (2090)	Significant improvement (*p* < 0.001)	Significant increase (*p* < 0.001)	Significant decrease (*p* < 0.001)	NR	Improved scores	Marked LUTS improvement, safe under local anesthesia; positive sexual outcomes.
Yang et al., 2023 [30]	8 (1015)	−11.37 pts	+5.26 mL/s	−13.18 mL	NR	No significant change	Sustained LUTS relief; sexual function preserved.
Manfredi et al., 2021 [31]	18 (NR)	Significant improvement	Significant improvement	Significant reduction	NR	No new dysfunction	Significant LUTS improvement; excellent safety profile.
Miller et al., 2020 [32]	4 (514)	−10 to −11 pts	+3 to +6 mL/s	NR	NR	No new ED	Significant long-term LUTS relief; preserves sexual function; low retreatment rate.

Abbreviations: IPSS, International Prostate Symptom Score; Qmax, maximum urinary flow rate; PVR, post-void residual; VV, void volume; IIEF-5, International Index of Erectile Function; ED, erectile dysfunction; EjD, ejaculatory dysfunction; LUTS, lower urinary tract symptoms; BPH, benign prostatic hyperplasia; NR, not reported; TUNA, transurethral needle ablation; TUMT, transurethral microwave thermotherapy; RCTs, randomized controlled trials.
